# How is parental psychological control associated with adolescent mental health in economically disadvantaged areas? The serial mediating role of psychological reactance and problematic smartphone use

**DOI:** 10.3389/fpsyt.2024.1458378

**Published:** 2024-08-20

**Authors:** Qiangqiang Li, Shuwen Wei, Zixiao Liu

**Affiliations:** ^1^ School of Educational Science, Jiangsu Second Normal University, Nanjing, China; ^2^ Department of Social Work, School of Sociology and Political Science, Shanghai University, Shanghai, China

**Keywords:** parental psychological control, psychological reactance, problematic smartphone use, mental health, economically disadvantaged areas

## Abstract

**Introduction:**

Adolescent mental health has been an issue of global concern, and the mental health of adolescents in economically disadvantaged areas may require additional research. The research delves into factors associated with economically disadvantaged areas’ adolescent mental health, employing family systems theory, self-determination theory, and psychological reactance theory. Specifically, the present study which was done in Jingzhou country, an economically disadvantaged area of Hunan Province, China, aimed to examine the link between parental psychological control and adolescent mental health, as well as the mediating roles of psychological reactance and problematic smartphone use in this relationship.

**Methods:**

A sample of 1300 Chinese middle school students (620 girls, mean age = 14.22 ± 1.29) completed self-reported measures of parental psychological control, psychological reactance, problematic smartphone use, and adolescent mental health.

**Results:**

Results showed that parental psychological control was negatively associated with adolescent mental health. Psychological reactance and problematic smartphone use mediated the association between parental psychological control and adolescent mental health, separately and serially.

**Discussion:**

The findings of the present study enrich the literature on parenting styles and adolescent mental health in economically disadvantaged areas, and this provides an intervention perspective to reduce the negative impact of poor parenting on adolescent mental health in economically disadvantaged areas.

## Introduction

Adolescent mental health has always been the focus of concern in the academic field (1–3). In the year 2020, the World Health Organization released a comprehensive set of guidelines titled “Guidelines for the Promotion of Adolescent Mental Health and Preventive Interventions.” These guidelines emphasize that adolescents are at a considerably high risk for developing mental health issues. With a large proportion of the adolescent population (approximately 16% of the world’s population), the current research recognizes that addressing the primary threats to adolescent mental health is essential for the successful achievement of sustainable development goals. Since the reform and opening-up policy, China’s economic development and various social undertakings have made significant progress, and the standard of living has greatly improved (4). However, due to the imbalance in development between urban and rural areas, as well as between the eastern, central, and western regions, a considerable number of adolescents in China still live in economically disadvantaged areas. Numerous studies indicate that children from economically disadvantaged families are more likely to experience physical health, cognitive development, and social-emotional issues than children from affluent families (5–7). Economic disadvantage can force people to live under the great pressure of social exclusion (8). For adolescents growing up in low-income families, their developmental environment is usually even more challenging, with inaccessible healthcare and welfare support, inadequate education opportunities and resources, unavailable mentors and models within their social networks, and frequent exposure to antisocial peer groups and temptations from illegitimate opportunities (9, 10). Therefore, examining the factors influencing the mental health of adolescents in economically disadvantaged areas is of great practical importance for enhancing the mental health level of this group.

The family environment, as the most fundamental factor in individual development, plays a significant role in adolescent mental health (11, 12). Parental psychological control (PPC), as an intrusive parenting style centered on inducing guilt, withdrawing love, instilling anxiety, and limiting individual autonomy (13), is closely linked to adolescent mental health (14, 15). The self-determination theory (SDT) posits that individuals require autonomy independence, and the satisfaction of this need is fundamental for healthy development (16, 17). Due to its direct hindrance and suppression of adolescents’ autonomy needs, parental psychological control often leads to maladaptive issues in adolescents (18, 19). In addition, In China’s collectivist culture, parents tend to exert more psychological control than in some Western nations, like the United States, due to the belief that “my child is my report card.” (20). Therefore, it is necessary to explore the adverse consequences of parental psychological control on Chinese adolescent mental health.

What are the mechanisms by which parental psychological control affects adolescent mental health? The present study suggests that psychological reactance may play a crucial role in mediating the relationship between parental psychological control and adolescent mental health outcomes. Numerous studies indicate a close relationship between parenting styles and adolescents’ psychological reactance (21, 22). Maladaptive parenting can bring psychological pressure to adolescents, leading them to feel that their freedom is being violated without proper channels, thus resulting in psychological reactance. The social-ecological diathesis-stress model emphasizes that stressful life events (such as negative parenting style) can activate individuals’ cognitive vulnerabilities, causing them to adopt more negative cognitive patterns or belief systems that distort self-perception and external environments, ultimately leading to negative adaptation outcomes (23). Parental psychological control, as a negative parenting style, prompts adolescents to combat it to fulfill their autonomy needs. During this process, adolescents are at high risk of developing negative cognitions, particularly psychological reactance (24). Therefore, parental psychological control may have a detrimental impact on the mental health of adolescents through the mediating role of psychological reactance.

Moreover, in the digital age, one of the side effects of parental psychological control includes problematic smartphone use among adolescents. Research suggests that parental psychological control can lead to a decline in psychological security among adolescents (25), increased social anxiety (26), and need frustration (27), all of which give rise to issues of problematic smartphone use. Moreover, problematic smartphone use can have negative impacts on the mental health of adolescents (28, 29). Therefore, parental psychological control may also have negative effects on the mental health of adolescents through the mediating role of problematic smartphone use (excessive use of smartphones with impairment to academic and social function). Therefore, this research investigates the correlation between parental psychological control and mental health, and the mediating roles of psychological reactance and problematic smartphone use in this association.

### The relationship between parental psychological control and mental health

Family systems theory highlights the significant influence of the family on the psychological development of adolescents (30). Adolescents who experience dysfunctional family system dynamics face not only the dynamic interactions among subsystems but also the influence of unstable and flawed new systems, which can negatively impact their mental health (31). Furthermore, economic hardship can exacerbate the negative effects of family dysfunction on the mental health of adolescents. For instance, research indicates that mothers’ and fathers’ reports of economic hardship were linked to adolescents’ reports of economic hardship. This, in turn, had a detrimental effect on the warmth between parents and adolescents while increasing parent-adolescent conflict (32).

Drawing from this theory, previous research has linked parental psychological control with mental health and found that parental psychological control influences adolescents’ depressive emotions to a certain extent, making children and adolescents prone to being overly sensitive in interpersonal interactions (33), as well as various addictive behaviors (34, 35). To our knowledge, however, the relationship between parental psychological control and mental health has not been thoroughly examined. Previous studies (36–39) have mostly explored the relationship between parental psychological control and adolescents’ negative mental health status (40, 41), while neglecting positive mental health indicators. According to Han et al. (2020) standards, positive indicators such as interests and self-esteem can measure positive mental health status, while negative indicators such as behavioral issues, low self-esteem, and depression can assess negative mental health status (42). Therefore, for a comprehensive evaluation of adolescents’ mental health issues, both positive and negative states need to be considered. Therefore, the present study aims to investigate the relationship between parental psychological control and mental health. Additionally, exploring the mechanisms underlying this relationship would also be valuable. In conclusion, this study proposes research hypothesis 1: parental psychological control will have an adverse impact on adolescents’ mental health.

### Psychological reactance as a mediator

Psychological reactance theory (43) posits that when something threatens or eliminates people’s freedom of behavior, they experience psychological reactance, a motivational state that drives freedom restoration. Psychological reactance is a common psychological phenomenon during adolescence (44). Research indicates that parental parenting styles are the primary predictive factors of children’s psychological reactance (45). Significantly positive correlations exist between parental psychological control, particularly over-control, excessive protection, refusal, and denial of children’s problematic behavior, and a significant reduction in children’s sense of social responsibility due to such refusal and denial (46). Similarly, the family environment inevitably influences adolescents’ psychological reactance. Studies have found that the lower the degree of autonomy adolescents have in the family, the stronger their psychological reactance is (47).

Self-determination theory suggests that individuals require autonomy independence, and the satisfaction of this need is the foundation for a person’s positive development (17). On the other hand, parental psychological control, as a negative parenting approach, hinders and suppresses the autonomy needs of adolescents. Research indicates that under the influence of psychological reactance, individuals often engage in behaviors contrary to the recommended action (such as increasing exercise frequency, adopting healthy eating habits, abstaining from alcohol, and refraining from smoking) (48). However, due to the “boomerang effects” of psychological reactance and the simultaneous desire to break free from parental psychological control, adolescents are susceptible to developing behaviors that harm their mental health (such as staying up late, reducing the frequency of social interactions, and experiencing low moods) (24). This can lead to decreased sleep quality among adolescents (49), poor interpersonal relationships (50), and negative emotions (51), ultimately resulting in compromised mental health. In conclusion, parental psychological control not only triggers psychological reactance in adolescents, but this reactance also indirectly results in mental health issues among them. Therefore, this study proposes research hypothesis 2: parental psychological control may have a negative impact on adolescent mental health through the mediating effect of psychological reactance.

### Problematic smartphone use as a mediator

According to Billieux and colleagues (52, 53), problematic smartphone use (PSU) is defined as “an inability to regulate one’s use of the smartphone, which eventually involves negative consequences in daily life”. Over the past decade, smartphone use has become widespread among today’s children and adolescents which parallels increases in poor mental health in this group (54, 55). There has been much recent research concerning the prevalence of problematic smartphone use in children and young people who use smartphones and how this syndrome relates to mental health outcomes (29). Among Chinese adolescents, problematic smartphone use ranges from 27.6% to 29.8% (56). Frequent smartphone use is associated with stress, depression, and sleep disturbances (57, 58).

On one hand, previous research has found that problematic smartphone use is influenced by family environmental factors (59, 60). Parental psychological control involves intrusive and manipulative parenting behaviors that are maladaptive. This negative parenting style can restrict children’s emotional experiences and expressions, undermining adolescents’ autonomy development (61). Empirical studies have found that adolescents’ perception of parental psychological control can significantly predict their problematic behaviors, such as problematic internet use (62) and internet gaming addiction (63). Thus, we have reasons to believe that parental psychological control can also predict adolescents’ problematic smartphone use behavior.

On the other hand, adolescent problematic smartphone use is a kind of behavior that leads to mental health issues. Research shows that different dimensions of problematic smartphone use are significantly negatively correlated with adolescents’ mental health. Firstly, there is a significant negative correlation between adolescents’ smartphone social networking and their mental health. Previous research has found a significant positive correlation between adolescent depression and the time spent on online social networking (64). Secondly, a significant negative correlation exists between middle school students’ smartphone entertainment gaming and mental health. For example, Andreassen et al. (2016) found that electronic gaming as a major form of online entertainment is associated with mental disorders such as anxiety, depression, loneliness, attention-deficit/hyperactivity disorder, and obsessive-compulsive disorder (65). In conclusion, not only does parental psychological control lead to adolescents’ problematic smartphone use, but problematic smartphone use also causes adolescents’ mental health problems. Hence, we posit hypothesis H3: parental psychological control may negatively affect adolescents’ mental health through the mediating role of problematic smartphone use.

### The serial mediating effect of psychological reactance and problematic smartphone use

Therefore, based on the hypothesis, what is the relationship between psychological reactance and problematic smartphone use during the mediation process? Psychological reactance occurs when individuals perceive their behavioral freedom as threatened, leading them to protect their choice and rebuild motivation for freedom. Psychological reactance increases the attractiveness of restricted behavior to individuals, driving them to repeat the behavior and manifest it through cognition, behavior, and emotions (43). With the convenience of smartphone use, adolescents are becoming increasingly dependent on their phones, prompting parental intervention in their usage. A previous study has shown a connection between parent-child relationships and adolescents’ problematic smartphone use; the more parental conflicts, the higher the level of psychological reactance in children, increasing the likelihood of adolescents developing smartphone dependency (66). Therefore, the present study suggests that adolescents with higher levels of psychological reactance are more inclined to assert their independence, making it difficult for them to accept parental restrictions on smartphone use, potentially triggering strong resistance motives and exacerbating smartphone dependency issues. Thus, this study proposes hypothesis 4: psychological reactance can positively predict problematic smartphone use, and there is a chain mediating effect of psychological reactance and problematic smartphone use in the impact of parental psychological control on adolescents’ mental health.

### The current study

**Figure 1:**
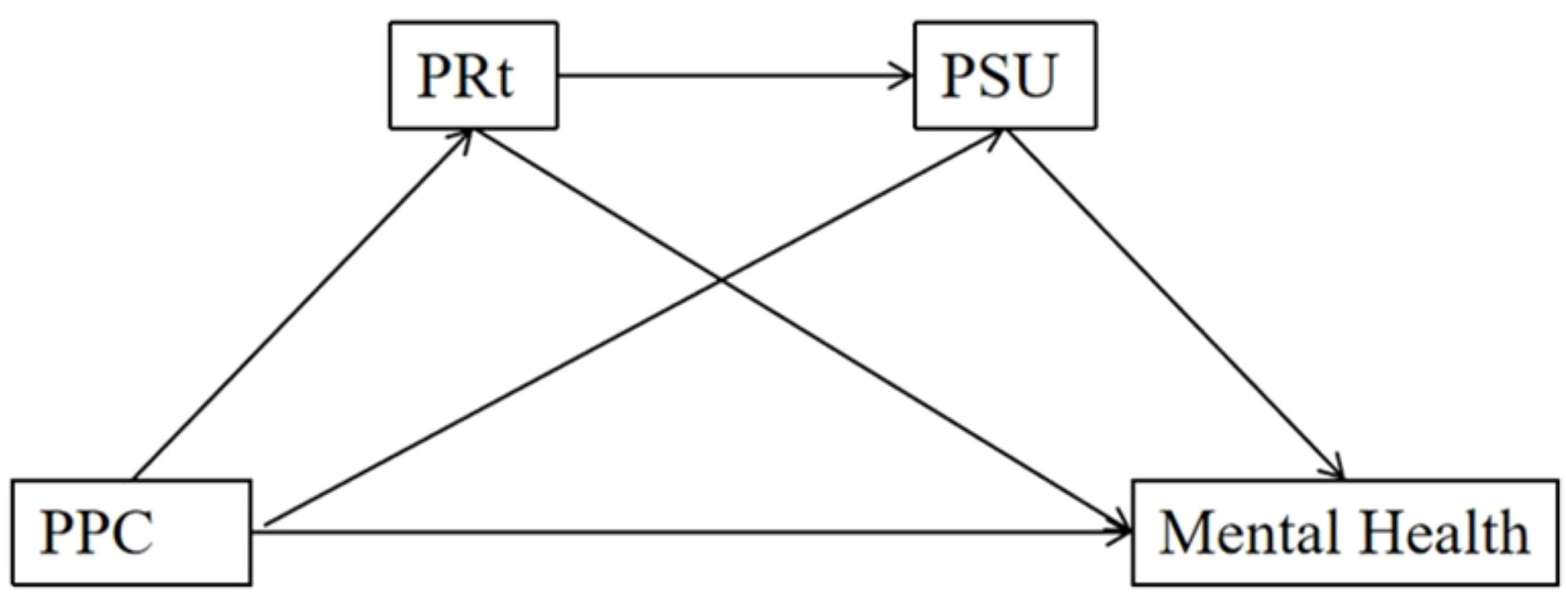
The proposed moderated mediation model. PPC, parental psychological control; PRt, psychological reactance; PSU, problematic smartphone use; →: the arrow signifies a predictive function.

The current study aims to systematically examine the impact of parental psychological control, psychological reactance, and problematic smartphone use on the mental health of adolescents in economically disadvantaged areas, as well as investigate the mediating roles of psychological reactance and problematic smartphone use. Please refer to **Figure 1** for more detailed information. This research not only elucidates the relationship and underlying psychological mechanisms between parental psychological control and the psychological health of adolescents in economically disadvantaged areas, but it also provides valuable insights for educators and parents in preventing and intervening in the mental health issues of adolescents in economically disadvantaged areas.

## Methods

### Participants

This research conducted a mental health survey in an economically disadvantaged area of Jingzhou in Hunan Province, China, in collaboration with the education department’s mental health cooperative project. A total of 1326 Chinese adolescents completed an anonymous self-report questionnaire voluntarily. After excluding participants who did not complete all questionnaire items, data for 1300 subjects were available for subsequent analysis. This study received approval from the local school ethics committee and obtained informed consent from school administrators, teachers, parents, and adolescents. The participants consisted of 620 females and 680 males, with 240 only being only children and 1060 non-only children, 1002 adolescents from two-parent families and 298 from single-parent families, 402 adolescents from rural areas, and 898 from urban areas. The average age was 14.22 years (*SD* = 1.29, range 12 to 17 years). All participants were of Han ethnicity and had used a mobile phone or computer for internet access in the past year.

### Measures

#### Parental Psychological Control

The Parental Psychological Control Questionnaire (67) in its Chinese version was used for measurement. A 5-point Likert scale was used to score 36 items (18 items for father’s psychological control and 18 items for mother’s psychological control). A higher average score indicates a stronger perception of PPC in adolescents. The questionnaire demonstrated satisfactory reliability and validity, making it suitable for evaluating the level of PPC perception in Chinese adolescents (67). In this study, the Cronbach’s alpha for the PPC questionnaire was 0.93.

#### Problematic Smartphone Use

To measure PSU, we utilized a brief version of the Smartphone Addiction Scale for Adolescents (68). It has demonstrated satisfactory reliability and validity among Chinese secondary school students (69). The tool consists of 10 items, rated on a 6-point Likert scale, ranging from 1 (strongly disagree) to 6 (strongly agree). The total score ranges from 10 to 60, with higher scores indicating problematic smartphone use. The questionnaire demonstrated sufficient internal consistency in this study (Cronbachcy Alpha = 0.93).

#### Psychological Reactance

The Hong’s Psychological Resistance Scale (HPRS; Hong & Faedda, 1996) (70) was utilized. It demonstrated satisfactory reliability and validity among Chinese high school students (71). Participants rated each item on a Likert 5-point scale, ranging from 1 (strongly disagree) to 5 (strongly agree). In this study, the revised HPRS showed a Cronbach’s alpha of 0.89.

#### Mental Health

The Mental Health Scale for Children and Adolescents (MHS-CA) developed by Cheng et al. <(>2006<)> (72) was used for measurement in Chinese adolescents. It demonstrated satisfactory reliability and validity in Chinese adolescents (73). The inventory encompasses 24 different components, segmented into 5 categories, namely Cognitive Processes and Language (encompassing thought processes, cognitive content, cognitive autonomy, language expression, and language comprehension with 5 items), Cognition (with perception, attention, memory, intelligence, learning and working with 5 items), Emotions (including emotional experiences, pleasant experiences, emotional reactions with 3 items), Volitional Behavior (comprising behavior, activities, interests, interpersonal interactions, health concerns with 5 items), and Personality Traits (involving self-confidence and self-esteem, safety and trust, sense of responsibility, liveliness, kindness, and need satisfaction with 6 items). Adolescents evaluated each component on a 7-point Likert scale, ranging from 1 (strongly disagree) to 7 (strongly agree). A higher average score correlates with better mental health. This scale not only covers negative aspects of mental health but also includes positive facets, enabling a more comprehensive assessment of adolescent mental health. In this study, the Cronbach’s alpha value was 0.91.

### Common variance test

Common method bias is the artificial covariation between predictor variables and criterion variables due to factors like the data source, measurement environment, project context, and project characteristics. To address this bias and enhance response authenticity, participants were assured anonymity and confidentiality in completing the questionnaire for academic research purposes. Analysis revealed eigenvalues of 14 factors exceeding 1 in total, with the largest factor explaining 25.35% of the variance (74). As the common method bias was below 40%, the study did not exhibit a significant common method bias effect.

### Preliminary analyses

**Table 1** presents the means, standard deviation, and Pearson correlations for all study variables. Parental psychological control was positively related to psychological reactance, and problematic smartphone use, and negatively related to mental health. Psychological reactance was positively associated with problematic smartphone use and negatively related to mental health. Problematic smartphone use has a negative relationship with mental health. In addition, age and gender had significant associations with study variables and were thus entered as covariates in the following analyses.

**Table 1:** Means, standard deviations, and correlations for main study variables.

Variables	*M*	*SD*	1	2	3	4	5	6
1. Age	14.22	1.29	1					
2. Gender	0.48	0.50	-0.01	1				
3. PPC	2.78	0.92	0.04	-0.26**	1			
4. PRt	2.99	1.50	0.07*	-0.04	0.43**	1		
5. PSU	4.27	0.63	0.10**	-0.04	0.32**	0.51**	1	
6. Mental health	3.37	0.55	-0.11**	0.01	-0.26**	-0.25**	-0.19**	1

N = 1300. *p < 0.05, **p < 0.01. PPC, parental psychological control; PRt, psychological reactance; PSU, problematic smartphone use.

### Testing for the mediation effects

**Figure 2:**
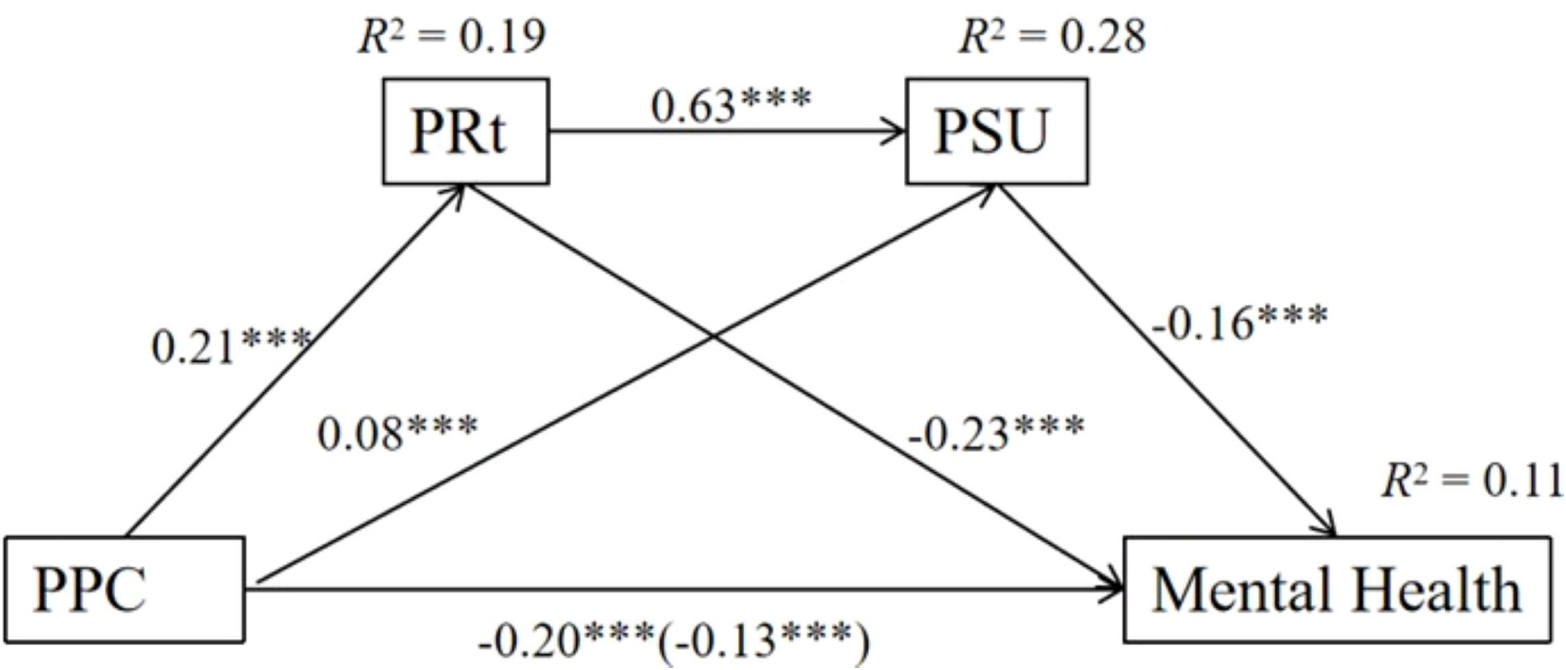
The Mediation Effects of psychological reactance and problematic smartphone use in the relation between parental psychological control and mental health. PPC, parental psychological control; PRt, psychological reactance; PSU, problematic smartphone use. →: the arrow signifies a predictive function. The number in the parenthesis is the regression coefficient for the total effect. *** *p* < 0.001.

To test the mediating effects of psychological reactance and problematic smartphone use (Hypotheses 2–4), Model 6 of the PROCESS macro (75) was adopted. As shown in **Figure 2**, parental psychological control was significantly and positively related to psychological reactance, *b* = 0.21, *p* < 0.001. Parental psychological control (*b* =0.08, *p* < 0.001) and psychological reactance (b =0.63, *p* < 0.001) were significantly and positively associated with problematic smartphone use. Furthermore, psychological reactance (*b* =-0.23, *p* < 0.001) and problematic smartphone use (*b* =-0.16, *p* < 0.001) were significantly and negatively related to mental health.

For the indirect effect, the results showed that the total indirect effect was 0.06 and significant, *SE* = 0.01, 95% CI = [-0.087, -0.039]. Specifically, there was a significant indirect path from parental psychological control to mental health through psychological reactance (indirect effect =0.04, *SE* =0.01, 95% CI = [-0.091, -0.033]), and a significant indirect path through problematic smartphone use (indirect effect =0.01, *SE* =0.00, 95% CI = [-0.016, -0.010]). The indirect path from parental psychological control to mental health through psychological reactance and problematic smartphone use serially was also significant (indirect effect =0.01, *SE* =0.01, 95% CI = [-0.019, -0.011]). Hypotheses 2, 3, and 4 were all supported.

## Discussion

### Parental psychological control and mental health

As predicted, our results revealed that parental psychological control was negatively associated with adolescent mental health. This finding aligns with the frameworks proposed by family system theory and self-determination theory, which emphasize the significant impact of family on the psychological development of adolescents. When adolescents encounter dysfunction within the family system, their mental health is compromised (31). Parental psychological control, as a prevalent form of family system dysfunction (76), parental psychological control drives parents to intrude into their children’s inner world, disregard their emotions, and restrict their self-expression. This detrimental parenting approach limits children’s emotional experiences and expressions, undermining their autonomy needs (61). Specifically, parental psychological control to some extent heightens adolescents’ depressive emotions, making children and adolescents overly sensitive in social interactions (33), and decreases positive emotions in adolescents, such as self-esteem and confidence levels (77, 78). Particularly, for adolescents in areas of economic hardship, economic stress has a direct effect on marital quality which, in turn, disrupts or undermines the parent-child relationship (79). Therefore, the current study integrates the positive and negative aspects of adolescent mental health to initially investigate the correlation between parental psychological control and adolescent mental health. Our result extends previous literature that examined the association between parental psychological control and adolescent mental health (80) and indicates that the relationship between appearance-related interaction on family parenting style and adolescent mental health is worth further investigating in future research.

### The mediating effect of psychological reactance and problematic smartphone use

As hypothesized, psychological reactance mediates the association between parental psychological control and adolescent mental health. This result is consistent with previous findings that psychological reactance scores correlated with parental punitive disciplinary styles (24, 81, 82). Although some previous studies have examined the associations among parental styles, psychological reactance, and emotional reactions and found that parent-child conflict is positively associated with psychological reactance, both of these two variables predicting negative emotional reactions (24), the mediating role of psychological reactance in the link between parental psychological control and comprehensive adolescent mental health is largely ignored. The present study thus innovatively examined and provided empirical evidence of this indirect effect. The findings of the mediation effect indicate that parental psychological control not only directly influences adolescent mental health, but also impacts it indirectly through its effect on adolescent psychological reactance. This is because adolescents have a need for autonomy. Parental psychological control hinders and suppresses adolescents’ need for autonomy, leading adolescents to easily develop psychological reactance (24) in order to break free from parental psychological control, ultimately resulting in compromised mental health. Given that psychological reactance specifically targets adolescents, it may be valuable to explore the impact of psychological reactance on adolescent mental health development in future research. Consistent with our hypothesis, problematic smartphone use mediated the association between parental psychological control and adolescent mental health. This result is in line with recent findings that problematic smartphone use serves as a mediator in the relationships between maladaptive parent-child relationships (e.g., parental neglect and parental psychological control) and negative outcomes, such as poor mental health (83–85). Specific to the present study, according to the self-determination theory, when adolescents perceive high levels of parental psychological control, it restricts their emotional experiences and expression, disrupting the autonomy development of adolescents. In order to meet the need for their own autonomy development and to seek freedom, adolescents often resort to higher frequencies of smartphone usage, including spending more time on social networks (64) and smartphone entertainment games (65). However, frequent use of online social networks and electronic games can lead to issues such as low self-esteem, anxiety, and attention deficits in adolescents, subsequently resulting in poor mental health.

In addition, the findings of this study showed that parental psychological control had an indirect association with adolescent mental health via the serial mediating effect of psychological reactance and problematic smartphone use. This serial mediating model advances our understanding of how parental psychological control is associated with adolescent mental health. In other words, when adolescents perceive parental psychological control, they develop a sense of protecting their autonomy and rebuilding motivation for freedom, known as psychological reactance. Adolescents exhibit psychological reactance in order to break free from parental control, restore emotional experiences and expressions, and meet their own needs for autonomous development. With the convenience of smartphone usage, parental interventions regarding adolescents’ phone use are becoming more pronounced. Under the influence of psychological reactance, smartphone usage increases its appeal to adolescents, leading to more severe problematic smartphone use. Moreover, adolescents experiencing psychological reactance may be more prone to conflicts with their parents. The more parent-child conflicts arise, the higher the level of adolescents’ psychological reactance, increasing the likelihood of developing smartphone dependency (66), ultimately resulting in more severe mental health issues among adolescents (54, 86). The serial mediating model in the present study suggests that psychological reactance might be included in the framework of self-determination theory when examining the link between interpersonal and parent-child experience to psychological problems. The role of psychological reactance in this theoretical framework needs to be further examined in future research.

### Implications and limitations

This study mainly has two points of implications. Firstly, the current study suggests that strong parental psychological control may be a risk factor for mental health among adolescents. Parents exerting excessive psychological control over their adolescent children could potentially lead to an increased risk of deterioration in mental health. This finding underscores the importance of parents reconsidering and decreasing their level of psychological control in order to mitigate such risks. In Chinese society, parental behavior is often influenced by the belief that their children’s accomplishments reflect their own self-esteem, leading to heightened psychological control compared to Western parenting practices (20). To address this issue, it is crucial to empower Chinese parents, enhance their self-worth, and promote a greater understanding of their children’s need for autonomy, ultimately reducing excessive psychological control. In addition, it can also help parents reduce their psychological control level by promoting a positive parent-child relationship (87). Therefore, the study identifies one key intervention strategy to curb the risk of mental health impairment among adolescents facing high levels of parental psychological control. Teaching emotional regulation techniques and promoting rational thinking is vital in alleviating negative psychological reactions. Additionally, given the cultural emphasis on filial piety and respect for elders in China, creating a supportive environment for adolescents to openly discuss their views and values can be beneficial (88). By fostering emotional expression and independence in Chinese adolescents, parents and educators can effectively reduce psychological reactance and encourage a healthier relationship dynamic. Secondly, another implication pertains to the serial mediation role of psychological reactance and problematic smartphone use in the context of parental psychological control and mental health outcomes. Consequently, the current study suggests that engaging in physical activities with parents might serve as an effective intervention strategy to mitigate the negative influences of parental psychological control on adolescents’ mental health. Through parent-child exercise, participating parents can impart knowledge, instill skills, and share experiences, thereby enhancing a positive parent-child relationship (89). Physical exercise inherently encompasses elements of enjoyment and competition, fostering greater interaction between parents and children and strengthening emotional ties. As a result, engaging in physical activities facilitates the establishment of robust parent-child relationships, which can significantly decrease adolescents’ levels of psychological reactance, proving highly beneficial for their mental health (90). Furthermore, physical exercise may assist teenagers in reducing their frequency of smartphone usage (91). In sum, appropriate parent-child physical activities can help adolescents alleviate the negative impact of psychological reactance and problematic smartphone use on their mental health.

Although theoretically supporting the proposed intervention measures, this study still has three limitations. First, the study used a cross-sectional method, suggesting a bidirectional association between parental psychological control and externalizing problems. Longitudinal studies and laboratory experiments can further explore the bidirectional association between parental psychological control and mental health. Second, the current study focuses on the relationship between parental psychological control and the mental health issues of adolescents in economically disadvantaged areas. Considering the universality of the problem, future research could incorporate relatively affluent regions in China to investigate how cultural differences influenced by varying economic backgrounds may affect parental psychological control. There may be significant disparities in parent-child relationships across different economic contexts, and such differences are likely to have varying impacts on adolescent mental health. It remains to be empirically validated whether psychological reactance and problematic smartphone use act as mediating factors in this relationship. Third, we only studied adolescents in the background of Chinese, where the family culture is unique (e.g., In China’s collectivist culture, parents tend to exert more psychological control than in some Western nations, like the United States, due to the belief that “my child is my report card.”); thus, we cannot guarantee the reproducibility of results in Western countries and encourage future research to directly test these questions in this cultural context.

## Conclusions

This study found that psychological reactance and problematic smartphone use, both separately and serially, mediated the negative relationship between parental psychological control and economically disadvantaged area’s adolescent mental health. The findings of this study extend the extant literature and family systems theory, self-determination theory, and psychological reactance theory by exploring the association between parental psychological control and adolescent mental health and the mediating roles of psychological reactance and problematic smartphone use in this relationship. Furthermore, our findings of the present study enrich the literature on parenting styles and adolescent mental health in economically disadvantaged areas, and this provides an intervention perspective to reduce the negative impact of poor parenting on adolescent mental health in economically disadvantaged areas. The current research addresses the direct effects of parental psychological control on adolescent mental health, as well as the serial mediating role of problematic smartphone use on adolescent mental health, proposing corresponding coping strategies. These strategies focus on enhancing parents’ emotional regulation techniques and promoting parent-child physical activities.

## Data Availability

The original contributions presented in the study are included in the article/supplementary material. Further inquiries can be directed to the corresponding author.
